# Evaluation of medicine retail outlets for sale of typhoid fever vaccine among adults in two urban and rural settings in western Kenya: a proof-of-concept study

**DOI:** 10.1186/s12913-016-1788-5

**Published:** 2016-09-29

**Authors:** Julius Ho, Gladys Odhiambo, Lucy W. Meng’anyi, Rosemary M. Musuva, Joseph M. Mule, Zakayo S. Alaly, Maurice R. Odiere, Pauline N. Mwinzi, Lisa Ganley-Leal

**Affiliations:** 1 Alpert Medical School, Brown University, Providence, RI USA; 2 Implementation Research Solutions (IRSK), Kakamega, Kenya; 3 Kenyatta National Hospital, Nairobi, Kenya; 4 Neglected Tropical Diseases Branch, Centre for Global Health Research, Kenya Medical Research Institute, P. O. Box 1578-40100, Kisumu, Kenya; 5 Epsilon Therapeutics, Inc., Newtown, USA

## Abstract

**Background:**

Private sector medicine outlets are an important provider of health services across the developing world, and are an untapped means of distributing and selling vaccines outside of childhood immunization programs. The present study assessed the viability of medicine outlets (chemists and pharmacies) as potential channels for sale of vaccines.

**Methods:**

To evaluate the viability of the medicine outlet model, we partnered with nine outlets across urban and rural communities in western Kenya to sell a nurse-administered typhoid vaccine. Purchasers were surveyed to reveal market demographic characteristics, reasons for vaccine purchase, and sources of information about the program. Key informant interviews and focus group discussions defined acceptability, demand, and additional suggestions for improving this mechanism of selling and distributing vaccines.

**Results:**

There was a higher than expected demand for the vaccine that resulted in stock-outs. Previous instance of typhoid, desire to prevent disease, affordable price and convenience were cited by most participants as main reasons for purchase of vaccine at the local outlet. The most common source of information on the vaccine sale was word-of-mouth and referral from friends. Longer vaccine sale duration, adequate stocking of vaccines and extended hours of administration in the evening to allow working individuals to buy vaccines were cited by participants as ways for improved participation in the future.

**Conclusions:**

This study demonstrated a high demand for vaccines at community medicine outlets. Important insights on how to improve and sustain such a program included extension of distribution time, education of outlet keepers, and minimizing vaccine stockouts. With improved social marketing, infrastructure mapping, education and pricing schemes, medicine outlets could become a sustainable avenue for selling adult vaccines in emerging markets for both routine and pandemic vaccines.

**Electronic supplementary material:**

The online version of this article (doi:10.1186/s12913-016-1788-5) contains supplementary material, which is available to authorized users.

## Background

The current immunization agenda heavily emphasizes childhood vaccination with the Global Vaccine Action Plan (GAVI) setting vaccination coverage target of ≥90 % nationally [1, 2]. Indeed, in most developing countries, immunization schedules are designed around the Expanded Program on Immunization-a set of essential vaccines to be given in infancy and early childhood [3]. The trend has been to integrate vaccine distribution into the delivery of maternal and child health services [4, 5] or in supplemental immunization campaigns and special child health days [6, 7]. These efforts have resulted in significant increases in childhood immunization rates.

In contrast, relatively little attention has been paid to vaccination among older populations. The immunization needs of older age groups is a major challenge 21^st^ century, as childhood coverage improves and rising life expectancy results in different, more chronic diseases assuming the global burden of disease [8, 9]. These include current and future vaccines against food-borne and sexually transmitted diseases, neglected tropical diseases, and emerging pandemics, which necessitate reaching a broad age range. Even in the developed world, adult immunization is considered lacking in terms of its outcomes, funding, and policy framework when compared to children [10–12]. Alternative models of vaccine distribution, which can reach older populations are needed to meet the needs of massive rollouts.

The private retail sector has recently played an important role in the USA, particularly for flu vaccination [13]. We thus hypothesized that the private sector could be tapped for increasing immunization rates in the developing world. The retail pharmaceutical sector includes a variety of chemists and pharmacies that are highly ubiquitous across both high and low income countries. These medicine outlets vary in their services, products and scope of practice, and some of the owners, in particular of chemists, often do not have any formal medical training or certification to act as bona fide healthcare providers [14, 15]. Nonetheless, outlet keepers (those manning outlets) frequently serve as a first point of medical consultation and dispense drugs and health products to millions of customers [16]. Because of their proximity, accessibility, and relationships with local communities, medicine outlets represent an important potential partner in immunization efforts for older populations or during emergency pandemics as a viable mechanism to generate revenue for research and development on much needed new vaccines.

This study was a proof-of-concept exploring the use of medicine outlets for the distribution and sale of vaccines. The objective of the study was to assess the viability of medicine outlets as potential channels for sale of vaccines geared toward adult populations. We recruited owners of chemists and pharmacies in peri-urban and rural western Kenya to sell a nurse-administered adult vaccine for typhoid fever, a local endemic disease. Customer and sales data were collected to determine the feasibility and potential demand for vaccination at these sites. This study demonstrates the practicality of developing the network of medicine outlets as relevant vaccine sales channels, thereby opening up markets as well as increasing accessibility.

## Methods

### Study setting

The study took place in western Kenya, within the cities of Kisumu (population 500,000; 30 % unemployment rate; 50 % absolute poverty) and Kakamega (population 600,000; 30 % unemployment rate, 57 % absolute poverty) where chemists and pharmacies are important and relatively well-documented sources of health services [17–20]. Both regions are known to have poor access to public health care facilities.

### Selection of medicine outlets

Any chemist or pharmacy that stocks and sells medicines was defined as a medicine outlet. Five outlets in Kakamega County were tested: one from each of three randomly selected urban informal settlements - Maraba, Amalemba, and Lurambi, and two in rural settings - Bushiri and Makunga. Four medicine outlets were chosen in Kisumu County: three from randomly selected urban informal settlements - Obunga, Nyalenda, and Manyatta, and one in a rural setting - Nyahera. The outlets were chosen based on willingness of the outlet owner/keeper to participate in the study and record purchasing data from clients.

Outlets were evaluated for 3 days prior to study in order to generate baseline data on the types of medicines purchased and overall volume of sales. Chemist owners and outlet keepers were trained in the collection of purchasing data before and during vaccine distribution using a mobile application on smartphones, enabling the collection and uploading of all field data on site. Open Data Kit (ODK) (Dept of Computer Science and Engineering, University of Washington) was used to build a data collection form, accessible by devices using the Android operating system, which uploaded responses in.xls format to an online database created with Google App Engine (Google Inc.- Mountain View, CA). The questionnaire on the smartphones gathered customer demographic information (name, age, gender, occupation), how customers heard about the study, and their reasons for purchase of vaccines.

### Community sensitization

A road show was utilized to advertise the vaccine campaign in public locations prior to the sale of the vaccines. A truck was affixed with banners promoting typhoid vaccine and equipped with a public address system, which broadcasted messages about the study. Team members accompanied the vehicle and answered questions from those who attended. The second component of publicity efforts took place at the selected chemists and pharmacies. Each site was provided with laminated posters and paper brochures containing information about typhoid vaccination.

### Sale and administration of typhoid vaccine

We selected a typhoid vaccine (Typhim Vi- Sanofi Pasteur, Lyon, France) as a prototypical product for vaccines for adults and which is not part of any childhood immunization programs. Vaccines were purchased from a supplier (Laborex Chemist- Kisumu, Kenya) at a market price of ~9.0 USD per dose, and were sold for a price of ~50 Kenya shillings (KSh) (~0.6 USD). The medicine outlet owners were allowed to keep all revenues from vaccine sales. Five experienced nurses in vaccination protocols who were registered with the Nursing Council of Kenya (NCK) were recruited to administer the vaccine. Nurses were trained on the use of a mobile phone platform for data collection. Following community advertising, nurses were stationed in the designated chemists and pharmacies, and administered the vaccine during business hours for a planned distribution period of up to 3 days. The vaccine was maintained centrally under refrigeration at IRSK offices where the nurses collected their daily supplies in temperature-monitored cooler boxes every morning, and returned any left over at the end of the day for cold storage. After receiving the vaccine, customers were issued official immunization certificates and asked to complete a short, oral questionnaire administered by the nurses and the opportunity to participate in a group discussion (described below). Chemists and pharmacies maintained normal operations during this time.

### Key informant interviews and focus group sessions

After administration of the vaccine, key informant interviews (KII) were conducted with outlet keepers and nurses who participated in the study. The interviewers encouraged free and open responses highlighting the challenges and opportunities encountered during vaccine distribution.

Three focus group discussions (FGD) with community participants were also conducted, including those who purchased the vaccine and those who did not purchase. These consisted of two groups of 8–12 participants per village, one consisting of males and the other females. Three primary topics were explored in the FGDs: 1) perceptions on the effectiveness of social mobilization tools in enhancing the knowledge of typhoid fever and its impact in increasing the demand and uptake of the vaccine; 2) factors that contributed to participation and non-participation of community members in the study; 3) willingness to pay for a vaccine and price points. We also gathered recommendations for future vaccine campaigns through medicine outlets.

### Analysis

Sales information collected from medicine outlets and customer surveys were input into spreadsheets. All KIIs and FGDs were tape recorded and transcribed. Textual data was fed into qualitative analysis software (ATLAS/ti 6.0- ATLAS.ti GmbH, Berlin, Germany) where it was coded with selected themes. A master sheet analysis was then carried out, using all the responses from the FGD and interviews. Responses were translated to English and interpreted by selecting patterns in the responses and formulating themes which could account for those responses. These responses were then presented in table format. Quantitative data formatting, standardization and analysis were carried out using both Excel and STATA 12 (Stata Corporation, College Station, TX.).

## Results

### Baseline outlet sales prior to vaccine campaign

Outlet keepers across the nine study sites recorded baseline data on a total of 1735 customer transactions. Two of the sites collected data over a period of four consecutive days, while the remainder collected data for 3 days. Baseline sales showed that the most common category of product purchased was analgesics (28.2 %), followed by antibiotics (24.2 %). Remaining categories were allergy medication (3.1 %), anti-malarials (9.6 %), other anti-microbials (6.2 %), cold medication (2.8 %), contraceptives (7.7 %), nutritional supplements (8.7 %) or other (9.5 %). Using the value of each transaction, we calculated summary statistics on all items (irrespective of category) to illustrate the typical range of costs for products sold in medicine outlets. The cheapest items tended to be analgesics, while prescription antibiotics constituted the most expensive purchases (Table 1).

**Table 1:** Price distribution of baseline transactions

Outlet #	Number of sales	Mean value	Median value	SD of value	Min value	Max value
1	190	58.22	40	68.36	5	570
2	150	27.07	15	26.21	5	130
3	235	65.32	40	99.25	5	800
4	139	85.18	50	95.81	10	540
5	155	65.52	50	59.17	10	300
6	290	40.99	30	42.13	2	350
7	79	51.03	50	30.12	8	100
8	277	62.96	50	57.88	10	560
9	220	93.47	80	67.51	6	400
All outlets	1735	61.32	49	68.86	2	800

### Vaccine customer characteristics

Data was collected on 613 customers at the time of vaccine administration. Vaccine purchase was equal between male (50.1 %) and female (49.9 %) participants, with a mean age of 35.9, median age of 32, and a standard deviation of 12.8 (Table 2). Customers were next asked to share their reasons for purchasing a vaccine. Affordable price was by far the most common reason, cited by 56.0 % of respondents (Table 3). Finally, customers were asked on how they heard about the vaccine sale exercise at their local outlet. The single most common source of information was word-of-mouth and referral from friends (46.8 % of respondents). Road shows (18.4 %) and promotional materials (brochures/posters) at medicine outlets (15.7 %), the two major publicity efforts, did not reach the majority of customers. Other sources, including announcements from outlet keepers and the internet, were cited by 25.8 % of respondents.

**Table 2:** Socio-demographic characteristics of vaccine customers

Description	Frequency (*n* = 613)	Percentage (%)
Gender		
Male	307	50.1
Female	306	49.9
Occupation*		
Businessperson	265	43.2
Farmer	64	10.4
Food handler	56	9.1
Teacher	41	6.7
Other or unemployed	201	32.8

*Total percentage exceeds 100 % because respondents reported multiple jobs

**Table 3:** Reasons for purchasing vaccine

Reason	Frequency (*n* = 613)	Percentage (%)
Affordable price	343	56.0
Typhoid prevention	229	37.4
General health	19	3.1
Availability	15	2.4
Past history of typhoid	12	2.0
Other	5	0.8

### Focus group results

#### Opinions about distribution and service at the medicine outlets

Participants were generally positive about the process of vaccine distribution. From the community perspective, most said they would still buy the vaccine if it was sold in the future. When asked about the difference between sites of sale, the medicine outlets versus hospital, the majority of participants cited convenience and cost as motivators for buying from a medicine outlet. Customers stated that hospitals were farther away, required long waiting times and medical consultation before vaccination, and charged more fees.

“*At the hospital, you have to plan. You decide ‘I will wake up at this time’ and so on, but at the chemist or pharmacy you can continue with your own business and access is easy.*” - adult male from Kakamega.

Many people indicated they were hesitant to visit hospitals for fear of queues and complications in service. Community chemists were often a more desirable venue to seek healthcare in general:

“*Chemists usually are in the centre of the community so most of the people rush there for prevention. I think one will seek help there first, when things are difficult is when you go to the hospital*” - adult female from Kisumu.

#### Reasons for purchase

The majority of focus group participants from the community cited a previous instance of typhoid, often multiple episodes in past years, as their main reason for buying the vaccine. The desire to prevent the disease was also mentioned by many who did not report a past infection. A common theme among participants was to rationalize the purchase of vaccine in economic terms:

“*I felt vaccination protection is better than cure, the cost of treatment is much higher than protection. Better to protect myself than to get the disease and seek treatment.*” - adult female from Kakamega.

All participants reported that they were very satisfied with the service provided by nurses in the exercise, both in how they educated customers and how they administered the vaccine. Some said the presence of a uniformed nurse made them feel more comfortable, evoking a hospital setting.

#### Affordability

Participants stated that the outlet price was significantly lower than the usual cost of receiving vaccine at hospitals - reported figures ranged from 400 to 1500 KSh - and compared favorably to the cost of treating typhoid. Nevertheless, a minority believed that 50 KSh would still present a challenge to some of the poorest (and especially rural) members of the community; a reduction to 20–30 KSh was suggested. Most participants believed that increasing the price of the vaccine, to 100 KSh or 200 KSh, would result in lower uptake. However, it should be noted that products purchased from the outlets were as high as 800 KSh (Table 1). Those willing to purchase a more expensive vaccine were predicted to be those who were acquainted with the cost of treatment.

#### Reasons for not purchasing a vaccine

Most of the reasons participants gave for not purchasing the vaccine were related to lack of access. More than half of the participants reported their key reason was the vaccine shortage. Due to greater than expected demand, medicine outlets were out of stock during publicized days of distribution, resulting in consumers being turned away. This was complemented by the common complaint that daytime hours of distribution were in conflict with consumer’s work schedule or other commitments. A few respondents were not aware of the distribution exercise until the material day, which presented a financial challenge or otherwise prevented them from making a purchase decision.

Outside of access, an important issue was consumer concern regarding whether the vaccine had been stored properly, and whether the staff was trained. Some consumers were cautious of the outlet keeper’s motives, noting that business owners may pay more attention to making vaccine sales than proper procedure. Others raised questions about the authenticity of the vaccine. Some participants also voiced concerns and suspicions about potential side effects, or that the injections were part of a sterilization/family planning scheme. For other participants, receiving the vaccine from a pharmacy was acceptable as long as the outlet was recommended or certified by the government, could demonstrate the vaccines came from a legitimate source, and had medical authorization to give them. Only a few participants said they would not buy vaccines from the outlet again, saying they would feel safer at the hospital with specialists. At least one community member appreciated the medical examinations which accompany vaccination at the hospital, and was skeptical that sped-up administration at the pharmacy was appropriate.

### Key informant interview results

#### Outlet keepers

All outlet keepers strongly felt that this vaccine distribution exercise was a worthwhile activity. Outlet keepers liked revenue generated from sales. Many expressed willingness to take on the role of administering vaccines in the future - one pointed out that some pharmacists already administer birth control injections - and would be comfortable with additional training.

#### Nurses

Being the only participants to have experienced the exercise at multiple sites, nurses reported differences in service provision between locations. One nurse observed one outlet keeper who did not actively mention the vaccine and exhibited condescending attitudes towards her customers of lower socio-economic status. Another nurse initially observed low participation in her setting, and after talking to the community realized the outlet keeper commanded little respect. In contrast, uptake was high at one site where the outlet keeper was noted to be very well informed and trusted. Some outlet keepers and nurses reported that a few customers misconceived the program to be a clinical trial of an experimental vaccine or fertility drug.

### Suggestions for improvements from all study participants

Though the reception to the exercise was largely positive, participants offered numerous suggestions for future vaccine distribution (summarized in Table 4). The majority felt that the timeframe was a barrier to access; the overall schedule (3 days) should be extended to at least 1 week. A lengthened campaign could also potentially mitigate overcrowding at the medicine outlets. At a minimum, participants felt that outlets should be stocked with sufficient inventory. Stockouts in the present study resulted in many sites concluding their sales prematurely and wasted customer time. Finally, extended hours of administration in the evening would allow working individuals to be vaccinated.

**Table 4:** Summary of Focus group suggestions on distribution, sensitization and publicity

Lengthen vaccine distribution period to at least 1 week	
Bring significantly more vaccines to each site	
Sell vaccine in the evenings after work hours	
More time between mobilization and delivery (at least 2 days’ notice)	
Liaise with the chief, bring up vaccine distribution at chief’s baraza (community gathering)	
Inform the local public health officers	
Post materials at schools (give to children) and in churches to reach different segments of pop	
Advertise on the radio	
Use community health workers for door-to-door outreach	

## Discussion

Robust participation in the exercise and feedback from focus group discussions suggest that selling vaccines through medicine outlets is a promising model for increasing vaccine distribution. Both the availability of vaccines through medicine outlets and subsidized prices increased accessibility to vaccines. Customers found the new location of vaccine administration to be advantageous with respect to accessibility and convenience. The barriers to vaccination we identified in the communities in western Kenya, including the distance to hospitals and long wait, echo the findings of vaccination studies in other settings around the world [21, 22]. Resources and time expended on travels negatively affect adult vaccine uptake. The optimal placement of distribution sites has been the subject of previous study [23]. Medicine outlets, which are embedded in the communities they serve, are well-situated to reduce the indirect costs of vaccination. It was also noted that hospitals already refer patients to pharmacies and sometimes medicine outlets to purchase medicine, lending them additional credibility in providing health services.

To better understand how this partnership with the local private sector may be generalized, it is worth considering the well-documented and successful use of private medical vendors in distributing anti-malarial medications in sub-Saharan Africa. The global effort to make artemisinin-based combination therapies, the current first-line treatment for malaria, less expensive and available at medicine outlets has been a widely hailed initiative that invites comparison: by utilizing the private for-profit sector, there have been large increases in the accessibility and use of a product which previously was limited to the clinical setting [24]. Many of the concerns expressed by participants in this study are closely related to previously identified barriers to malaria treatment [25]. The policy implications of involving retail pharmacies in malaria control - adherence to treatment guidelines, quality assurance, and social responsibility of using profit-seeking entities - have also been explored in literature [26, 27]. For any expansion of vaccination through medicine outlets, similar aforementioned policy issues will need to be the subject of more in-depth studies.

One of the few benefits to seeking care at hospitals was the reassurance provided from being seen at a formal institution. Likewise, the study nurses were effective because their uniforms and official qualifications created trust in the community. If outlet keepers could be trained on safe storage, injection, and potential side effects, the majority of customers would likely be willing to receive vaccines directly from them. Development of this vaccinator training program would require navigating local legal frameworks and input from Ministries of Health. Also worth considering are the many regulatory challenges introduced by broadening vaccine sales, such as registered inventory, inspection of facilities, certification of personnel, and enforcement. The literature on malaria control again offers insight on these issues, and provides examples of successful interventions to improve retailer practices and engage them in broader government health initiatives [16, 28, 29].

Interestingly, we found that word-of-mouth was the most powerful source of information to inform communities of the vaccine program. The role of peers in disseminating information is a type of “social learning” that influences health behavior in developing countries [30, 31]. Restructuring our program to utilize this process - by increasing the lead time between publicity and the start of vaccine sales, and by lengthening the period of distribution - would enable information about the campaign and people’s experiences to disseminate further. In this way, community sensitization could be improved without much additional expenses.

The low price of the highly subsidized vaccine in this study was a major asset in creating high customer demand for vaccination in outlets. The vaccines were sold for 50KSh, compared to a typical price of 400–1500 KSh for hospital-sold vaccines. The 50Ksh price was comparable to the mean price of other items sold. Most participants felt that this was affordable, and that increasing the price would result in lower demand, although pricing could vary depending on settings. The household willingness-to-pay for typhoid vaccine has been previously described in other settings, and findings from the present study are consistent with the view that vaccines are products exhibiting price elasticity [32]. If the model in the present study was ever generalized, medicine outlets would play a role in setting vaccine prices; however, the vendor-optimal price for maximizing profits may not be in line with the social goal of maximizing vaccine uptake. More research is thus needed to elucidate the balance between affordability and profit incentives for outlet keepers in each particular setting and the public health need for vaccines outside of childhood immunization programs [33]. One barrier to vaccine distribution is poor electricity infrastructure in most rural settings in developing countries that would significantly impact on storage of vaccines and affect the cold-chain process required for most vaccines.

Considering the inadequate vaccine inventory during the exercise, reliable data collection should be considered a core function essential to supply chain management and the logistics of any sustainable distribution network for vaccines [34]. Data collection application tools that can be readily used by nurses and other portable technologies can play important roles in effectively delivering vaccines from suppliers to populations of need.

## Conclusion

It is important to recognize that the medicine outlet model is not a panacea for achieving universal vaccine coverage, but rather one to increase the supply chain for adult and adolescent vaccines, including mass rollouts of newly introduced vaccines, and would need to be supplemented by additional efforts to reach remote populations. Important insights on how to improve and sustain such a program included extension of distribution time, education of outlet keepers, and minimizing vaccine stockouts. With improved social marketing, infrastructure mapping, education and pricing schemes, medicine outlets could become a sustainable avenue for distributing vaccines in emerging markets for both routine and pandemic vaccines. Navigating some of the policy considerations of incorporating outlet owners in national health strategies and training a workforce in vaccine administration would also improve the capacity of national health systems to address future public health emergencies.

## Additional files

Additional file 1: Outlet keeper baseline data on the types of medicines purchased, customer characteristics and overall volume of sales. (XLSX 114 kb) Additional file 2: Vaccine purchase data. (XLSX 41 kb) Additional file 3: Focus Group Discussion guide for Community member. (DOCX 14 kb)

## Abbreviations

FGD: Focus group discussion
GAVI: Global Vaccine Action Plan
KII: Key informant interview
KSh: Kenya shilling
ODK: Open Data Kit

## References

[1] Clements CJ, Nshimirimanda D, Gasasira A (2008). Using immunization delivery strategies to accelerate progress in Africa towards achieving the Millennium Development Goals. Vaccine.

[2] Clemens J, Holmgren J, Kaufmann SH, Mantovani A (2010). Ten years of the Global Alliance for Vaccines and Immunization: challenges and progress. Nat Immunol.

[3] World Health Organization vaccine-preventable diseases: monitoring system. 2013 global summary. Available from: http://apps.who.int/immunization_monitoring/globalsummary/. Accessed 26 Jun 2015.

[4] Wallace A, Dietz V, Cairns KL (2009). Integration of immunization services with other health interventions in the developing world: what works and why? Systematic literature review. Trop Med Int Health.

[5] Wallace AS, Ryman TK, Dietz V (2012). Experiences integrating delivery of maternal and child health services with childhood immunization programs: systematic review update. J Infect Dis.

[6] Doherty T, Chopra M, Tomlinson M, Oliphant N, Nsibande D, Mason J (2010). Moving from vertical to integrated child health programmes: experiences from a multi-country assessment of the Child Health Days approach in Africa. Trop Med Int Health.

[7] Igarashi K, Sasaki S, Fujino Y, Tanabe N, Muleya CM, Tambatamba B, Suzuki H (2010). The impact of an immunization programme administered through the Growth Monitoring Programme Plus as an alternative way of implementing Integrated Management of Childhood Illnesses in urban-slum areas of Lusaka, Zambia. Trans R Soc Trop Med Hyg.

[8] Duclos P, Okwo-Bele J, Gacic-Dobo M, Cherian T (2009). Global immunization: status, progress, challenges and future. BMC Int Health Hum Rights.

[9] Rappuoli R, Mandl CW, Black S, De Gregorio E (2011). Vaccines for the twenty-first century society. Nat Rev Immunol.

[10] Poland GA, Belmin J, Langley J, Michel JP, Van Damme P, Wicker S (2010). A global prescription for adult immunization: time is catching up with us. Vaccine.

[11] Wu LA, Kanitz E, Crumly J, D’Ancona F, Strikas RA (2013). Adult immunization policies in advanced economies: vaccination recommendations, financing, and vaccination coverage. Int J Public Health.

[12] Samb B, Desai N, Nishtar S, Mendis S, Bekedam H, Wright A, Hsu J, Martiniuk A, Celletti F, Patel K, Adshead F, McKee M, Evans T, Alwan A, Etienne C (2010). Prevention and management of chronic disease: a litmus test for health-systems strengthening in low-income and middle-income countries. Lancet.

[13] Russo T. Pandemic vaccine distribution policy for the twenty-first century. Homeland Security Affairs. 2012;8(4). Available at: https://www.hsaj.org/articles/207. Accessed 27 Sept 2016.

[14] Lowe RF, Montagu D (2009). Legislation, regulation, and consolidation in the retail pharmacy sector in low-income countries. Southern Med Review.

[15] Wafula FN, Miriti EM, Goodman CA (2012). Examining characteristics, knowledge and regulatory practices of specialized drug shops in Sub-Saharan Africa: a systematic review of the literature. BMC Health Serv Res.

[16] Wafula FN, Goodman CA (2010). Are interventions for improving the quality of services provided by specialized drug shops effective in sub-Saharan Africa? A systematic review of the literature. Int J Qual Health Care.

[17] Ruebush TK, Kern MK, Campbell CC, Oloo AJ (1995). Self-treatment of malaria in a rural area of western Kenya. Bull World Health Organ.

[18] Goel PK, Ross-Degnan D, McLaughlin TJ, Soumerai SB (1996). Influence of location and staff knowledge on quality of retail pharmacy prescribing for childhood diarrhea in Kenya. Int J Qual Health Care.

[19] Amin AA, Marsh V, Noor AM, Ochola SA, Snow RW (2003). The use of formal and informal curative services in the management of paediatric fevers in four districts in Kenya. Trop Med Int Health.

[20] Liambila W, Obare F, Keesbury J (2010). Can private pharmacy providers offer comprehensive reproductive health services to users of emergency contraceptives? Evidence from Nairobi, Kenya. Patient Educ Couns.

[21] Kim D, Lauria DT, Poulos C, Dong B, Whittington D (2014). Effect of travel distance on household demand for typhoid vaccines: implications for planning. Int J Health Plann Manage.

[22] Jeuland M, Marc J, Marcelino L, John C, Dale W (2010). Estimating the private benefits of vaccination against cholera in Beira, Mozambique: a travel cost approach. J Dev Econ.

[23] Kim D, Lauria DT, Whittington D (2012). Selecting optimal prices and outpost locations for rural vaccination campaigns. Int Reg Sci Rev.

[24] Tougher S, Ye Y, Amuasi JH, Kourgueni IA, Thomson R, Goodman C, Mann AG, Ren R, Willey BA, Adegoke CA, Amin A, Ansong D, Bruxvoort K, Diallo DA, Diap G, Festo C, Johanes B, Juma E, Kalolella A, Malam O, Mberu B, Ndiaye S, Nguah SB, Seydou M, Taylor M, Rueda ST, Wamukoya M, Arnold F, Hanson K, ACTwatch Group (2012). Effect of the Affordable Medicines Facility--malaria (AMFm) on the availability, price, and market share of quality-assured artemisinin-based combination therapies in seven countries: a before-and-after analysis of outlet survey data. Lancet.

[25] Chuma J, Okungu V, Molyneux C (2010). Barriers to prompt and effective malaria treatment among the poorest population in Kenya. Malar J.

[26] Kamat VR, Nyato DJ (2010). Soft targets or partners in health? Retail pharmacies and their role in Tanzania’s malaria control program. Soc Sci Med.

[27] Goodman C, Kachur SP, Abdulla S, Bloland P, Mills A (2007). Drug shop regulation and malaria treatment in Tanzania--why do shops break the rules, and does it matter?. Health Policy Plan.

[28] Goodman C, Brieger W, Unwin A, Mills A, Meek S, Greer G (2007). Medicine sellers and malaria treatment in sub-Saharan Africa: what do they do and how can their practice be improved?. Am J Trop Med Hyg.

[29] Rowa Y, Abuya TO, Mutemi WK, Ochola S, Molyneux S, Marsh V (2010). Factors influencing implementation of the Ministry of Health-led private medicine retailer programmes on malaria in Kenya. BMC Public Health.

[30] Dupas P (2011). Health behavior in developing countries. Ann Rev Econ.

[31] Leonard KL, Adelman SW, Essam T (2009). Idle chatter or learning? Evidence of social learning about clinicians and the health system from rural Tanzania. Soc Sci Med.

[32] Do GC, Whittington D, Le TK, Utomo N, Nguyen TH, Poulos C, Dang TD, Kim D, Nyamete A, Acosta C (2006). Household demand for typhoid fever vaccines in Hue, Vietnam. Health Policy Plan.

[33] Mobisson-Etuk LN (2011). *E*ffects of AMFm on retail prices of and pricing practices for ACTs and dominant anti-malarials in Kenya: early observations under the AMFm.

[34] Kaufmann JR, Miller R, Cheyne J (2011). Vaccine supply chains need to be better funded and strengthened, or lives will be at risk. Health Aff (Millwood).

