# Supplementary material for: Evaluation of medicine retail outlets for sale of typhoid fever vaccine among adults in two urban and rural settings in western Kenya: a proof-of-concept study
Source: BMC Health Serv Res. 2016 Sep 29;16:527. doi: 10.1186/s12913-016-1788-5 (PMC5043612; doi:10.1186/s12913-016-1788-5)
Supplement: Additional file 3: — Focus Group Discussion guide for Community member. (DOCX 14 kb) [file 12913_2016_1788_MOESM3_ESM.docx]

## FOCUS GROUP DISCUSSION GUIDE FOR COMMUNITY MEMBERS

**TITLE: INVESTIGATING THE POTENTIAL FOR MEDICINE SHOPS [KIOSK] TO PROMOTE AND DISTRIBUTE VACCINES**

**Who to interview:**  Half to be those who got the vaccine and half those who didn’t.

Good day. I am … … and my colleagues are …………. We are from …….. and we are here to get your views about the just ended vaccination that took place in the shops in your village. The information you provide will be used to improve on the delivery of the vaccines in future interventions and also to inform policy markers. We have invited you because of your experience in this community and the confidence we have in you. We will talk to you for about 30-45 minutes. Participation in this discussion is voluntary. Your name and what you say to us during this discussion will be kept confidential.

In this discussion, every answer within the study subject is correct, because it is your view on the subject of discussion. Every opinion is important and should be freely expressed. What we will learn from you today will be useful in the future in strengthening vaccine delivery systems. We wish to request your permission to take notes and to tape record the conversation so as to ensure that we do not miss or misinterpret any of your views after the discussion.

Do you have any questions about the study? YES/NO (**Moderator to record questions and answers provided**)

Are you willing to participate in the discussion?

**If No, thank the respondent and terminate the interview.**

**Community Perceptions**

1. Did you get vaccines that were being sold in the shops?

Probe for

- Reasons why they got vaccinated
- Reasons why not

2. What is your opinion about the price that was charged

Probe for

- Affordable
- Expensive

3. What is your feeling about the health care provider who gave the vaccine

Probe for

- Attitude of the health care provider

4. If another vaccine was to be sold in the same shop would you buy it?

5. If the vaccine was to be distributed by a Community Health Worker would you still buy it?

6. What did you like or not like about the whole process

Probe for

- Likes
- Dislikes

7. How did you get to know that there were vaccines in the shops?

Probe for

- Mass media: Radio
- Brochures, posters and other printed materials
- Professionals: Health workers; Teachers
- Community Health workers
- Family, friends, neighbours and colleagues
- Leaders: At Barazas; Religious leaders
- Other (please explain):

8. What is the community expectation of Future vaccine distribution?

9. Any Other Observation or Comment.
